# Splenogonadal fusion: a case report of three cases and a literature review

**DOI:** 10.3389/fped.2023.1269879

**Published:** 2024-01-04

**Authors:** Na Luo, Qitao Xu, Hao Wang, Jiahong Su, Shoulin Li

**Affiliations:** ^1^ Department of Radiology, Shenzhen Children’s Hospital, Shenzhen, China; ^2^ Department of Urology and Laboratory of Pelvic Floor Muscle Function, Shenzhen Children’s Hospital, Shenzhen, China

**Keywords:** splenogonadal fusion, testicular tumor, intra-abdominal cryptorchidism, orchiectomy, scrotal swelling

## Abstract

**Purpose:**

This case report aims to enhance the understanding of clinical physicians regarding splenogonadal fusion (SGF) and to help them consider SGF as a differential diagnosis when testicular tumors are suspected, thus avoiding unnecessary orchiectomies.

**Methods:**

We report three cases of SGF admitted to our hospital, one of which presented as a suspected testicular tumor. We also searched the literature on scrotal masses from the last 25 years and summarize the characteristics of cases of SGF manifesting as scrotal swelling combined with our cases.

**Results:**

After conducting a thorough search, we found a total of 24 publications relevant to this case study, which included 25 testes. All lesions were located on the left side, and the average age of those affected was 20.22 years. Seven cases were of the continuous type. Three cases presented with pain, all of which were intratesticular masses. Thirty cases had a definite onset duration, ranging from 3 weeks to 10 years. Nine patients (36%) underwent orchiectomy, and one underwent partial orchiectomy.

**Conclusion:**

It is crucial to identify SGF in the clinic. When a patient presents with scrotal swelling, diagnosing SGF preoperatively is challenging, and it should be considered when there is a long history of a stable testicular mass. An intraoperative frozen section should be performed if a testicular tumor is suspected to avoid unnecessary orchiectomy.

## 1 Introduction

Splenogonadal fusion (SGF) is a congenital malformation in which the spleen and gonads fuse during embryogenesis (1). SGF has two types, a continuous type and a discontinuous type, distinguished according to whether there is a connection between the two organs. Clinical physicians usually discover SGF accidentally through laparoscopic testicular exploration and preoperative ultrasonography, which reveals a laparoscopic testis attached to the spleen (2). Although there have been several reports using laparoscopic staged Fowler-Stephen (FS) surgery for SGF combined with impalpable testis, to our knowledge, we are the first to successfully detach the spleen and correct the testis using single-stage FS. When SGF presents as scrotal swelling, especially the discontinuous type, preoperative diagnosis of SGF is difficult, and some patients have undergone unnecessary orchiectomy due to misdiagnosis of SGF as a malignant tumor (3). Combining our cases with the relevant literature retrieved, we summarize the characteristics of SGF. Hopefully, this case report can enhance the understanding of this condition among doctors in urology, ultrasound, radiology, pathology, and other related departments, so they can make more accurate diagnoses and avoid overtreatment.

## 2 Case presentation

**Figure 1:**
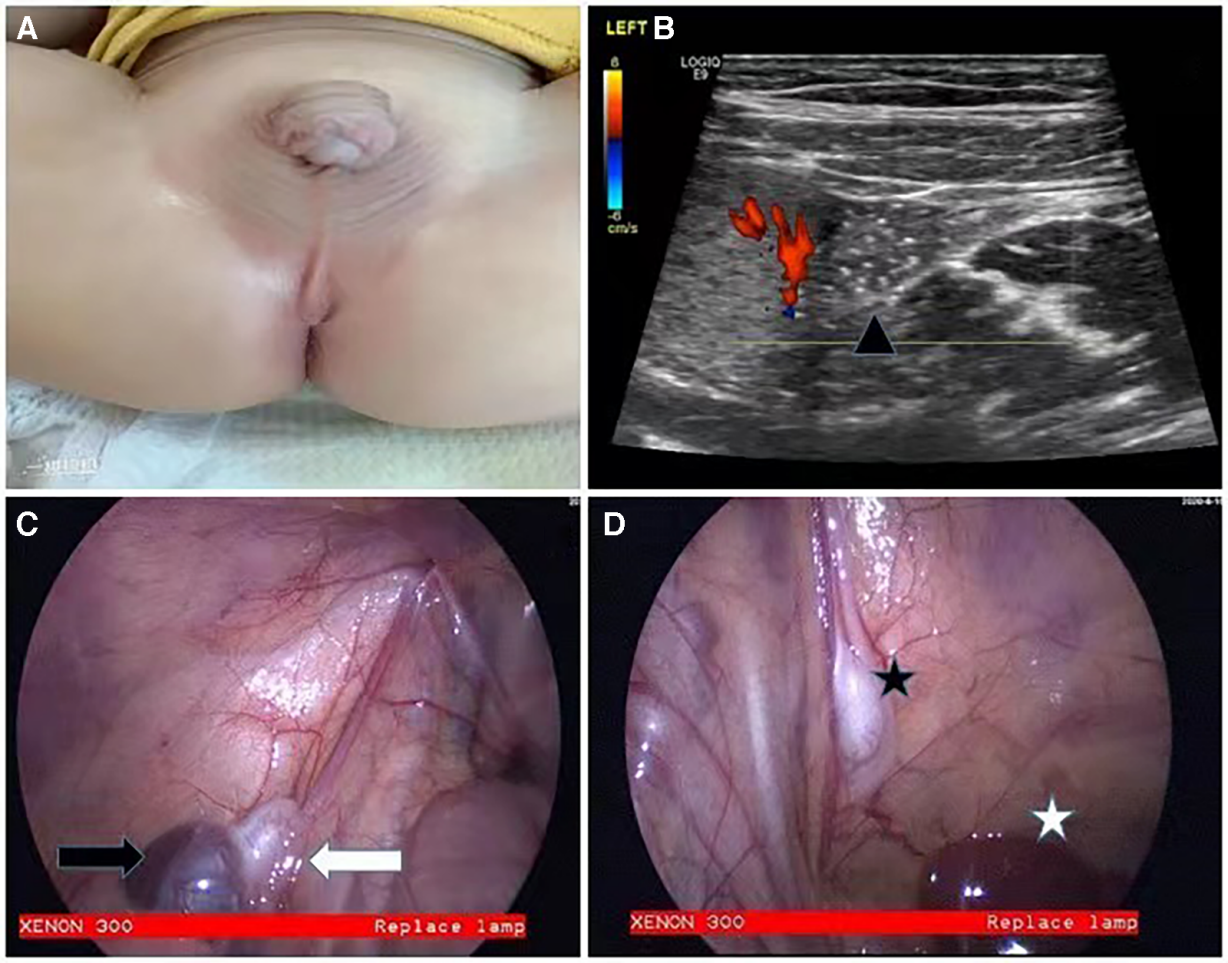
Appearance of case 1 and intraoperative findings. Physical examination showed bilateral impalpable testes and coronal hypospadias (**A**). Preoperative ultrasound suggested that the left testis was adjacent to the lower pole of the spleen (**B**). The left testis was fused to the spleen (**C**), and the right testis was located in a flat umbilical position at the lower edge of the liver (**D**) (Black triangle: left testis; black arrow: spleen; white arrow: left testis; black star: right testis; white star: liver.)

Case 1: A 20-month-old boy was admitted to the hospital for bilateral cryptorchidism. He had previously undergone atrial septal defect repair at the age of 8 months. Physical examination revealed bilateral impalpable testes and coronal hypospadias (Figure 1A). Preoperative ultrasound suggested bilateral intra-abdominal cryptorchidism, with the left testis positioned adjacent to the lower pole of the spleen (Figure 1B). Laparoscopic exploration revealed that both internal rings were closed, the left testis was fused with the spleen, and the diagnosis was continuous SGF (Figure 1C). The right testis was located in a flat umbilical position (Figure 1D), the gubernacular cord and vas deferens were thinly vascularized, and there was a possibility of bilateral testicular atrophy; furthermore, the urethra needed an additional operation for repair. Considering all these circumstances, the patient was considered to need three to four operations. However, the parents refused further surgery and withdrew from care, despite being informed in detail of the risk of testicular tumors.

**Figure 2:**
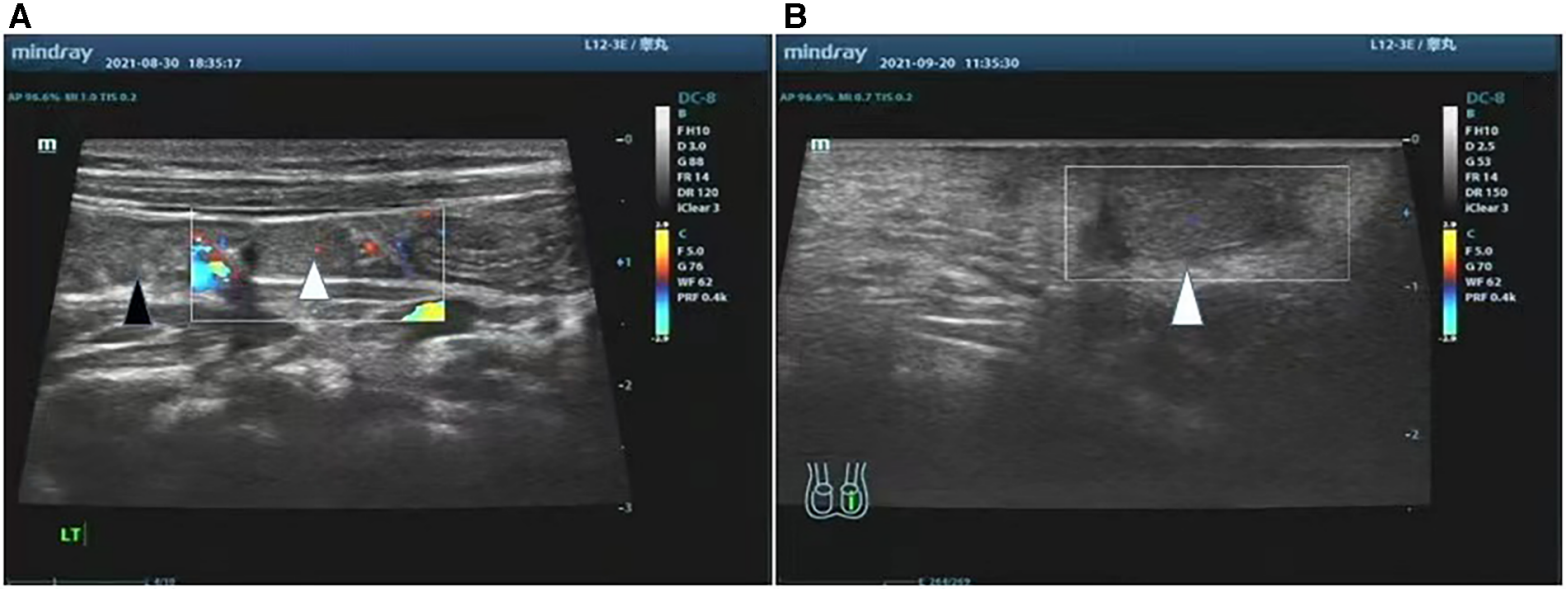
Pre-operative ultrasound and post-operative ultrasound of case 2 US examination of the left testis, it indicated the left testis was adjacent to the lower pole of the spleen (**A**). 1 month after surgery, the left testis was well positioned and survived (**B**). (Black triangle: spleen, white triangle: left testis).

Case 2: A 1-year-old boy was admitted to the hospital for bilateral cryptorchidism and hypospadias (penoscrotal type). Preoperative ultrasound suggested that the boy had SGF on the left side (Figure 2A). A single-stage Fowler–Stephens orchiopexy (FSO) was performed. After confirmation of the viability of the left testicle (Figure 2B), the boy underwent another single-stage FSO to bring the right testicle down into the scrotum. This procedure was performed 9 months after initial diagnosis. Four months later, the boy underwent repair of hypospadias. Ultrasound at this time showed that both testicles were in a good position and were viable.

**Figure 3:**
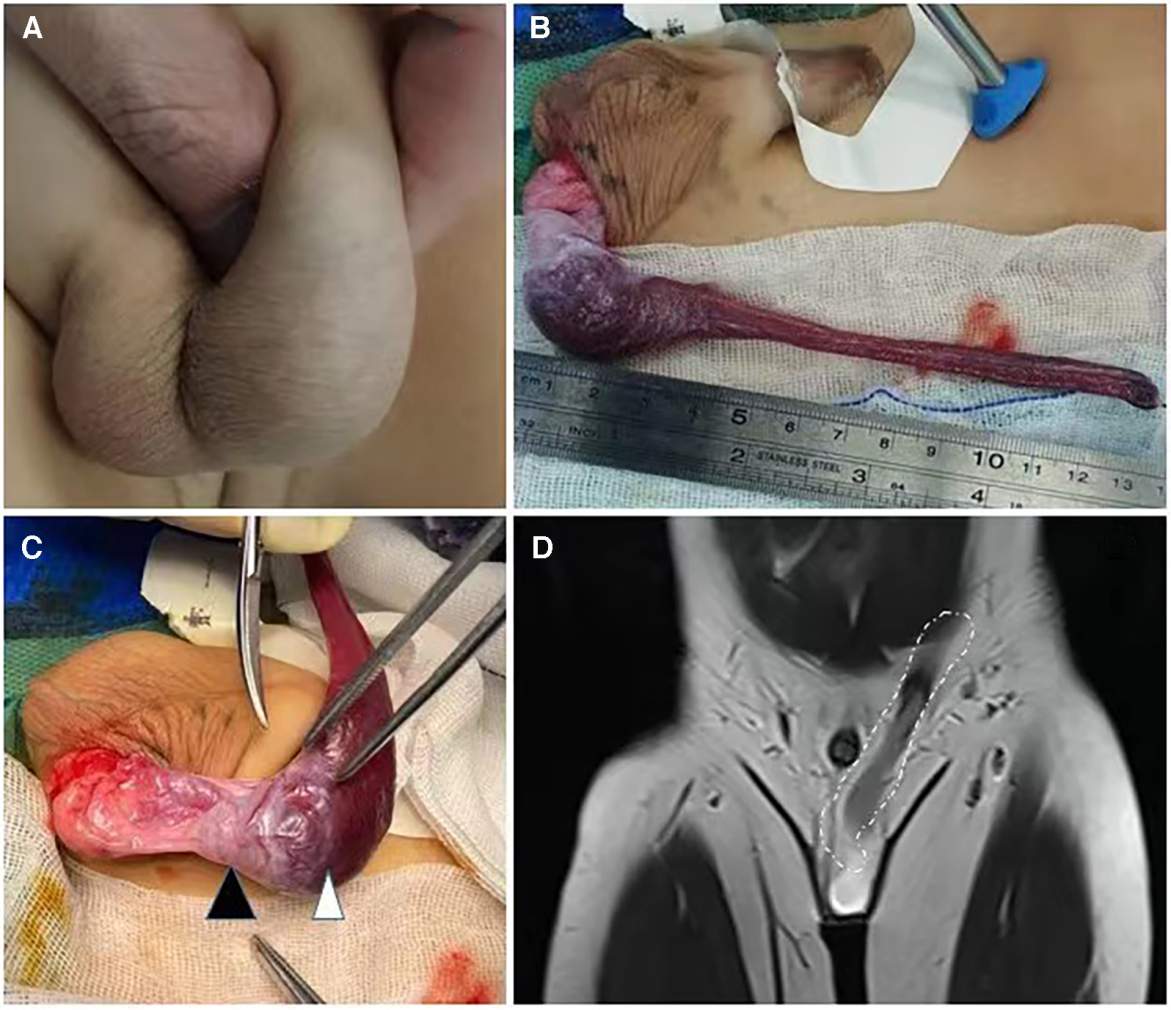
The appearance of case 3 and intraoperative findings On palpation of the scrotum, there was a firm mass located in the upper pole of the left testis (**A**). MR showed a cordlike structure extending from the upper pole of the mass to the abdominal cavity (**D**). The cord was ligated at a high position, and a transverse incision was taken in the left scrotum (**C**). The left testis and the connected splenic cord were pulled out completely (**B**), and the accessory spleen was removed and the testis was preserved. (White dotted circle: splenic cord, black triangle: left testis, white triangle: spleen)

Case 3: A 34-month-old boy was admitted to the hospital with painless enlargement of the left scrotum persisting for 2 years, with a recent slight increase in size; outpatient ultrasound revealed a testicular tumor. Physical examination suggested that, in addition to the cord-like mass in the upper pole of the left testis, a small amount of fluid could be found around the gonad (Figure 3A). There was no abnormality in the penis. After admission, the results of blood tests for tumor markers were normal. The outpatient ultrasound revealed only testicular tumors, with no hydrocele or inguinal hernia. Prior to conducting a pelvic enhanced MRI, we consulted with the radiologist to discuss any specific considerations that needed to be taken into account. During the examination, the radiologist found that the upper edge of the lesion entered the pelvic cavity along the left groin and eventually connected with the spleen (Figure 3D). Since the child was unable to cooperate with a lengthy MRI examination, no other sequences or cross-sections were added, but this observation can essentially explain the issue. Afterward, we arranged for a re-examination ultrasound. As we all know, the most common testicular tumor is teratoma, and the typical procedure for addressing it involves an inguinal or scrotal incision. This was also the procedure that we communicated to the patient’s parents prior to surgery. However, after the completion of pelvic MRI, we changed our minds based on the previous case of splenogonadal fusion. Continuous SGF was confirmed by laparoscopic exploration. A high ligation of the cord was performed, followed by a transverse incision in the left scrotum (Figure 3B). The left testis and the attached splenic cord were fully extracted, and the accessory spleen was removed while preserving the testis (Figure 3C). The presence of a fibrous capsule provides a clear demarcation between the splenic tissue and the normal testicular parenchyma, thus facilitating easier separation. Postoperative pathology confirmed the spleen. Ten days after the operation, the volume of the bilateral testes was mostly symmetrical, and the blood supply and the spleen in the abdominal cavity were good. The ultrasound was repeated three months later, and the results were the same as at the previous examination.

## 3 Discussion

The mechanism of splenogonadal fusion is not yet fully understood. The prevailing theory is that the genital crest and spleen primordium adhere or fuse during embryonic development. During the downward migration of the gonads, cases are divided into the continuous type and discontinuous type due to the breakage of fiber cords (4). It has also been suggested that splenic progenitor cells fuse with the primitive gonads via the retroperitoneal pathway, which could explain the phenomenon of right-sided non-contiguous splenogonadal fusion. Since the condition was first reported in 1883, approximately 230 cases have been reported; most of these cases were detected intraoperatively or at autopsy, and the rate of SGF detection is expected to increase with the availability of laparoscopic techniques. The male-to-female ratio is approximately 16:1, which may be an overestimate given that the male gonads are more superficial and easily examined. SGF is often combined with cryptorchidism, hypospadias, congenital heart disease, limb hypoplasia, micrognathia, and cranial anomalies. In our patients, the first two cases were combined with hypospadias and cryptorchidism. However, we performed diagnostic laparoscopy for impalpable testes, and SGF was diagnosed intraoperatively. Therefore, endocrine examination was not performed before surgery, even though karyotype analysis revealed a karyotype of 46XY. Fewer than 10 cases of SGF combined with cryptorchidism and hypospadias have been reported. Whether there is a relationship between SGF, cryptorchidism, and hypospadias requires more research.

There have been several cases of successful treatment of splenogonadal fusion combined with intra-abdominal cryptorchidism (5). However, SGF manifesting as scrotal enlargement is still mistaken for malignancy in many cases, ultimately leading to unnecessary orchiectomy. Improving awareness of the disease also makes our preoperative diagnosis more precise and avoids intraoperative changes during surgery, thus serving the best interests of the patient and their family. To this end, we searched the literature and identified 24 relevant publications (Table 1) in the last 25 years, focusing on diagnosis and treatment of SGF where testicular tumor was suspected (3, 6–28). The 24 cases in the literature plus the one case reported herein bring the total number of documented cases to 25, all left-sided, with a mean age of 20.22 years. Three cases presented with pain. We excluded one case from this count of presentations with pain because the lesion was a painless mass that had persisted for a long time before the patient was admitted with sudden pain. The remaining cases presented with painless scrotal swelling. There were 13 cases of clearly recorded duration, ranging from 3 weeks to 10 years, with no obvious change or a slight increase in swelling. Of the 25 cases, 7 (28%) were of the continuous type. In 32% of cases (8/25), the mass was located in the testis, including one case with two masses, one inside and one outside the testis (20). SGF was suspected before operation in only seven cases, of which five were of the continuous type. Finally, nine cases (36%) underwent orchiectomy, and one underwent partial orchiectomy (26). SGF patients usually have a distinct history in cases presenting as testicular tumors. The SGF mass persists for a relatively long time and takes a more variable course than testicular tumors, with fewer comorbidities, and only two of the 25 cases were combined with congenital malformations.

**Table 1:** General information on the cases retrieved from the literature.

	Age (years)	Symptom	Duration	Type	Prediagnosis	Location	Treatment
Ferrón and Arce (6)	2	PM	2 months	Dis	No	Extra	TTS
Jayasundara et al. (8)	0.42	PM	2 months	Con	Yes	Extra	TTS
Kocher et al. (7)	35	PM	UN	Dis	No	Intra	OM
Liu et al. (9)	6	PM	UN	Con	Yes	Extra	TTS
Bal et al. (10)	20	Sudden pain	UN	Con	No	Intra	OM
Chiaramonte et al. (11)	12	PM	UN	Dis	No	Intra	TTS
Sountoulides et al. (12)	31	Infertility	UN	Con	No	Extra	TTS
Zhou et al. (13)	9	PM	UN	Dis	No	Extra	TTS
Harris (14)	55	PM	UN	Dis	No	Intra	OM
Li et al. (15)	2	PM	UN	Con	Yes	Extra	TTS
Uglialoro et al. (16)	45	PM	10 years	Dis	No	Extra	TTS
Karray et al. (17)	38	PM	1 month	Dis	No	Extra	OM
Shakeri et al. (18)	4	PM	UN	Dis	No	Extra	OM
Grosu et al. (19)	53	PM	UN	Dis	Yes	Extra	TTS
Mann and Ritchie (20)	22	Pain	3 months	Dis	No	Intra and extra	OM
Seager et al. (21)	25	PM	many years	Dis	No	Extra	Biopsy
Guney et al. (3)	0.33	PM	3 months	Con	Yes	Extra	TTS
Kadouri et al. (22)	45	Pain	5 years	Dis	No	Intra	TTS
Patil et al. (23)	14	PM	3 years	Dis	No	Extra	OM
Daghas et al. (24)	3	PM	2 years	Dis	Yes	Extra	TTS
Fadel et al. (25)	31	PM	3 weeks	Dis	No	Extra	OM
Alkukhun et al. (26)	15	PM	1 year	Dis	No	Extra	Partial OM
Hartman et al. (27)	27	Pain	UN	Dis	No	Intra	OM
Kerkeni et al. (28)	8	PM	UN	Con	No	Extra	TTS

PM, painless mass; UN, unknown; Con, continuous; Dis, discontinuous; Extra, extratesticular; Intra, intratesticular; OM, orchiectomy; TTS, testicular sparing.

It is relatively challenging to distinguish the discontinuous type of SGF from testicular tumors because it often appears as a painless mass with a negative transillumination test, while the continuous type of SGF may show cord-like mass on physical examination. Laboratory tests are non-specific. Ultrasound is helpful in differentiating the two types of SGF. The continuous type usually emerges as a homogeneous mass entering the scrotum through the groin to the pelvis. In contrast, the non-continuous type usually presents as a low-reflectivity mass and may contain multiple small hypoechoic nodules, which may be associated with peak development of the lymphatic system and splenic white marrow in younger children; as a result, it has a higher diagnostic value in children aged 1–5 years who develop this condition (21). High vascular density in color Doppler flow imaging (CDFI) is helpful in the differential diagnosis of malignant tumors, and contrast-enhanced ultrasonography is also valuable in identifying benign and malignant testicular tumors (19). CT and MRI have high diagnostic value for continuous splenogonadal fusion. Sometimes, they can reveal the fibrotic tissue structure connected to the spleen and the left testis. However, no characteristic features have been observed in discontinuous splenogonadal fusion. ^99m^Tc sulfur colloid SPECT can reveal splenic tissue in the scrotum, but surgical exploration cannot be avoided, and histological examination is still required for final confirmation of the diagnosis (3, 23). As for continuous splenogonadal fusion, we believe that laparoscopic exploration should be performed.

After confirmation of the diagnosis of SGF, the splenic cord should be resected at a high position, and the patent processus vaginalis should be ligated simultaneously to avoid hernia, splenic torsion necrosis, or internal hernia. We chose to pull out the testis and splenic cord through the scrotum, which produces better aesthetic results (avoiding the inguinal incision) and can prevent the occurrence of iatrogenic cryptorchidism. In the case of an exploratory procedure for testicular tumor, an intraoperative frozen section should be performed simultaneously to avoid radical orchiectomy. There is also controversy as to whether the pars splenium should be removed, with a report of partial preservation of the spleen due to concerns about testicular blood supply, resulting in swelling of the accessory spleen during follow-up (29). In a case of SGF presenting as a testicular tumor, it was reported that dynamic observation was continued only after a biopsy of the mass. Subsequently, the diagnosis of discontinuous splenogonadal fusion was made (18). A fibrous capsule can be clearly observed between the testes and the spleen in both the continuous and the discontinuous types. This observation is based on previous publications that have showcased specimens and pathological sections (1, 12). However, most physicians choose to remove the spleen, not only because it is easy to detach the mass from the testis, but also because of the possibility of simultaneous pathological changes in the pars splenic tissue and normal splenic tissue, as reported in two cases of viral infection causing persistent painless swelling of the spleen in the scrotum (6, 8). In some hematologic diseases, such as hemolytic jaundice, where splenectomy is mandatory, all paratenic spleens should be removed together during surgery.

Interestingly, all three patients reviewed in this paper who presented with pain had masses in the testes, presumably due to local compression (10, 22, 27). In addition, long-term compression of spleen tissue may cause infertility, as shown by a 25-year-old patient whose puncture biopsy confirmed the absence of spermatogenesis in the left testis (30). Despite reports of SGF combined with testicular tumors, the patients in all five of these cases had a history of cryptorchidism, which is a known risk factor for testicular cancer, and there is no established relationship between SGF and testicular cancer (31).

## 4 Conclusion

Increased awareness of SGF is crucial. SGF presenting as cryptorchidism is often diagnosed by preoperative ultrasound or intraoperatively. Laparoscopic FS staged or single-stage surgery can achieve a satisfactory result. Preoperative diagnosis of SGF manifesting as scrotal swelling is complex, and clinical physicians should consider SGF when the patient has a long history of a stable testicular mass. We recommend laparoscopic high ligation of the splenic cord, ligation of the processus vaginalis, and transscrotal separation of the splenic cord from the testis to deal with the continuous type. Transinguinal incision is better for the discontinuous type, and intraoperative biopsy is needed to avoid unnecessary orchiectomy. Regarding the decision to keep or remove the accessory spleen, performing a transinguinal incision for mass excision is recommended. This is because an extratesticular mass can be easily separated from the testis or spermatic cord, while an intratesticular mass may compress the testis, causing pain or testicular atrophy.

## Data Availability

The raw data supporting the conclusions of this article will be made available by the authors, without undue reservation.
